# Cellular Response of Human Osteoblasts to Different Presentations of Deproteinized Bovine Bone

**DOI:** 10.3390/ma15030999

**Published:** 2022-01-27

**Authors:** Pedro Henrique de Azambuja Carvalho, Sarah Al-Maawi, Eva Dohle, Robert Alexander Sader, Valfrido Antonio Pereira-Filho, Shahram Ghanaati

**Affiliations:** 1 Department of Diagnosis and Surgery, School of Dentistry, Sao Paulo State University (Unesp), Araraquara 14801-060, Brazil; pedro.azambuja@unesp.br (P.H.d.A.C.); dinho@foar.unesp.br (V.A.P.-F.); 2 FORM-Lab, Frankfurt Oral Regenerative Medicine, Department for Oral, Cranio-Maxillofacial and Facial Plastic Surgery, Medical Center of the Goethe University, 60323 Frankfurt, Germany; sarah.Al-Maawi@kgu.de (S.A.-M.); Eva.Dohle@kgu.de (E.D.); r.sader@em.uni-frankfurt.de (R.A.S.)

**Keywords:** deproteinized bovine bone matrix (DBBM), guided bone regeneration, purification, cellular inflammatory response

## Abstract

*Objectives*: This study evaluated the cellular response of primary osteoblasts exposed to two different presentations of a low-temperature non-sintered deproteinized bovine bone matrix (DBBM). *Materials and methods*: Six different baths of a commercially available DBBM block (Bonefill^®^ Porous Block) and one of DBBM granule (Bonefill^®^ Porous) were evaluated to identify the mineral structure and organic or cellular remnants. Samples of the same baths were processed in TRIZOL for RNA extraction and quantification. For the immunologic cell reaction assay, primary human osteoblasts (pOB) were exposed to DBMM block (pOB + B) or granules (pOB + G), or none (control) for 1, 3, or 7 days of cell cultivation. Expression of proinflammatory cytokines by pOB was evaluated by crosslinked ELISA assay. In addition, total DNA amount, as well as cell viability via LDH evaluation, was assessed. *Results*: Organic remnants were present in DBBM blocks; 45.55% (±7.12) of osteocytes lacunae presented cellular remnants in blocks compared to 17.31% (±1.31) in granules. In three of five batches of blocks, it was possible to isolate bovine RNA. The highest concentration of TGF-β1 was found in supernatants of pOB + G on day 7 (218.85 ± 234.62 pg/mL) (*p* < 0.05), whereas pOB + B presented the lowest amount of TGF-β1 secretion at the end of evaluation (30.22 ± 14.94 pg/mL, *p* < 0.05). For IL-6 and OPG, there was no statistical difference between groups, while pOB + G induced more IL-8 secretion than the control (3.03 ± 3.38 ng/mL, *p* < 0.05). Considering the kinetics of cytokine release during the study period, all groups presented a similar pattern of cytokines, estimated as an increasing concentration for IL-6, IL-8, and OPG during cultivation. Adherent cells were observed on both material surfaces on day 7, according to H&E and OPN staining. *Conclusion*: Neither tested material induced a pronounced inflammatory response upon osteoblast cultivation. However, further studies are needed to elucidate the potential influence of organic remnants in bone substitute materials on the regeneration process.

## 1. Introduction

The use of bone substitute material to support bone regeneration is a common purpose in regenerative medicine and oral surgery. There are numerous commercially available materials which are indicated for bone regeneration procedures [1,2]. They exhibit different chemical composition, physicochemical structure, and mechanical properties, and they undergo various producing or purification processes [3,4]. Regarding their origin, the bone materials can be autologous, allogeneic, xenogeneic, synthetic, or alloplastic [5,6].

Each type of biomaterial demands a particular processing method. For allografts and xenografts, the purification process is essential to remove the organic remnants of the appropriate donor, which potentially carry pathogens, proteins, or foreign genetic material which can lead to disease transmission or exacerbate an inflammatory reaction [7,8]. Bone substitute materials should accomplish a series of requirements to be suitable for clinical use. In addition to advantageous properties such as osteoconductivity or osteoinductivity of a material, biocompatibility is one of the most important requirements [9,10,11,12].

The incorporation of the graft and the formation of new bone is mediated by numerous cellular and molecular pathways that promote and regulate the activity of osteoblasts and osteoclasts [13,14,15]. Therefore, bone substitutes must fulfill several requirements. They should serve as a scaffold for the cellular migration of osteoblasts and endothelial vessel formation. In addition, they need to be noncytotoxic, compatible with human cells, and hydrophilic, s well as have similar mechanical resistance to the host bone [13].

Regarding the cytotoxicity and inflammatory reaction, purity has been considered a good parameter to evaluate the suitability of biomaterials. Non-autologous bone materials should ideally contain a pure mineral structure, without organic or antigenic remnants [7,8]. However, some commercially available purification methods for allografts or xenogeneic bone materials present some degree of organic remnants. This purity was classified by Ghanaati et al. [8] in five levels ranging from 0 (no organic remnants and no lamellar bone structure) to 4 (material containing donor cellular remnants in the trabecula).

The current purification methods described implicate complex physical and chemical steps to free the material from immunogenic components and attend to safety requirements. Nevertheless, despite the efforts for a standard purification process, some naturally derived bone materials still contain cellular or organic remnants in their composition [7]. In addition, materials that have been proven to be free of organic components might lose their lamellar structure as the purification process changes their physicochemical properties [8,14,15].

Bone substitutes have been applied for several medical indications, for example, to treat fractures, to maintain bone structure, and to support oral and facial bone regeneration [6,11,13]. However, as most clinical studies that evaluated bone substitute materials focused on new bone formation or bone maintenance, less is known regarding the biology of the interaction between bone substitutes and the receptor site, which could be affected by the biological properties of each biomaterial. However, there is still a lack of information about how the manufacturing, the purification process, and the purity degree affect the cellular response to bone substitutes.

The aim of this study was to characterize different forms of deproteinized bovine bone matrix (DBBM) (Bonefill^®^ Porous Block/Granules, Bionnovation, Bauru, Brazil), purified only by chemical process, and to analyze the in vitro cellular response of human primary osteoblasts (pOB) when exposed to the biomaterials. Therefore, osteoblasts were evaluated for their morphology, cell viability, and cytokine and growth factor release in response to the different biomaterials.

## 2. Materials and Methods

### 2.1. Material Origin

Bonefill^®^ Porous (Bionnovation, Bauru, Brazil) is a commercially available DBBM in chips and blocks, derived from the bone of bovine femur. According to the information obtained by the manufacturer’s manual inside the product pack, Bonefill^®^ is obtained by “crushed fresh bone submitted to a sequence of baths that solubilize the organic structures such as cells remaining from the organic matrix, fibers, and proteins, leaving only the mineral portion, which is sterilized through gamma radiation (25 kGy)”, according to the manufacturer. The biomaterial originates from tracked Brazilian herd, declared free of bovine spongiform encephalopathy (BSE) according to the International Zoosanitary Code and Scientific Seeing Committee of the European Union (SSCEC of August 2005). Furthermore, the manufacturer states that the purification process does not submit the bovine bone to high-temperature treatment. 

### 2.2. Biomaterial Characterization Ex Vivo

#### 2.2.1. Sample Preparation

Two different forms of biomaterials were evaluated in this study. The first group included Bonefill^®^ Porous Block (B) (Bionnovation, Bauru, Brazil), while the second group included Bonefill^®^ Porous granules (G) (Bionnovation, Bauru, Brazil). Five different batches of each biomaterial form were randomly obtained directly from manufacturer at two different timepoints. Under sterile conditions, the samples were divided into two parts: one for histological analysis and another for RNA extraction. Furthermore, different batches of each biomaterial were used for cell culture experiments [7,8].

#### 2.2.2. Histological Analysis

For histological analysis, samples were treated as previously described [7,8]. In brief, the samples were decalcified in 10% Tris-buffered EDTA solution at 37 °C for 7 days. Following decalcification, samples were dehydrated in a series of increasing alcohol concentrations and xylene in a preprogrammed tissue processor (TP1020, Leica Biossytems Nussloch Gmb, Nußloch, Germany) and embedded in paraffin blocks. Using a rotatory microtome (Leica M2255, Wetzlar, Germany), seven slides of 3–5 µm thickness were obtained from the most central part of material and prepared for hematoxylin and eosin (H&E) and Azan trichrome histological staining. In addition, tartrate-resistant acid phosphatase (TRAP) histochemical staining was performed to assess the possible TRAP expression. The histological analysis was performed with a Nikon Eclipse 80i light microscope (Nikon Europe b.v, Amsterdam, The Netherlands) to evaluate the macro and microstructure of the biomaterial, as well as the arrangement and possible presence of organic components. A microscope-connected DS-Fi1 Digital camera and a DS-L2 digital sigh control unit (Nikon, Tokyo, Japan) were used to scan and digitalize slides. From each sample, the H&E stains were used to identify osteocyte lacunae and the presence of cellular remnants. The total bone lacunae and the lacunae with cellular remnants were counted in the NIS-Elements software (Nikon Instruments Inc., Melville, NY, USA) at a 100× magnification. Azan trichrome was used to qualitatively assess the bone matrix and the presence of collagen remnants, while TRAP staining was used to determine the presence of possible osteoclasts or multinucleated giant cells (TRAP^+^ cells).

#### 2.2.3. RNA Extraction

Samples for total RNA extraction were transferred to 2.0 mL cryogenic tubes and immersed for 5 min at liquid nitrogen. Samples were removed from cryogenic tubes with the aid of a sterile forceps, placed in a sterile plastic covering, and smashed with a hammer, all under sterile and RNAase-free conditions. The powder obtained from this process was placed in a new sterile 1.5 mL tube, and the total RNA extraction and purification was performed using 1 mL of TRIZOL reagent (Sigma-Aldrich, Brøndby, Denmark) added to each sample and incubated for 15 min at room temperature, and then for 2 h at 4 °C. Briefly, purification was performed by adding 200 µL of chloroform (Sigma-Aldrich, Brøndby, Denmark) to each tube following by 10 s of vortexing and incubation at room temperature for 15 min. Tubes were centrifuged at 12,000× *g* for 15 min at 4 °C before the aqueous phase (transparent phase) containing RNA was transferred to a new 1.5 mL tube. After adding 500 µL of isopropanol, the tubes were incubated at room temperature for 10 min for RNA precipitation and then submitted to a new centrifugation step (12,000× *g*, 4 °C, 10 min), after which the supernatant was removed, and a pellet containing RNA was formed. The pellet was submitted to a DNase digestion step with 2 µL of DNase I stock solution (Qiagen RNase-free DNase set, Quiagen, Hilden, Germany), 10 µL of RDD buffer (Quiagen, Hilden, Germany), and 87.5 µL of RNase-free water (RFW, Quiagen, Hilden, Germany). After 10 min of incubation at 37 °C, DNase digestion was stopped by adding 50 µL of TRIZOL reagent and 50 µL of chloroform, mixed through pipetting. The solution was centrifuged (7200× *g*, 10 min, 4 °C), and the transparent phase was added to 250 µL of absolute ethanol and 10 µL of 3M sodium acetate in a new tube incubated a −20 °C for 90 min. Tubes were centrifuged (max speed, 10 min, 4 °C), before supernatant was removed and 75% ethanol was added to each pellet and centrifuged again (max speed, 5 min, 4 °C). The supernatant was removed, and pellets were left to dry on air inside a sterile hood. Dried pellets were resuspended with 11 µL of RFW, and RNA concentration was measured using a nanodrop spectrophotometer (NanoDrop, Wilmington, NC, USA).

### 2.3. Human Osteoblast (pOB) Response In Vitro

#### 2.3.1. Primary Cell Culture

Primary cells used for this study were obtained from excess tissue in accordance with the principle of informed consent and approved by the responsible Ethics Commission of the State of Hessen, Germany.

Primary human osteoblasts (pOB) were isolated and cultured according to a previously described protocol [16,17]. Briefly, excess tissues from the surgery room were obtained from different donors who did not present any health conditions affecting bone metabolism. After bone osteotomy, bone fragments which would otherwise be discarded were transferred to cell culture medium containing 1% penicillin/streptomycin and taken to the cell culture lab. Bone fragments were minced and placed in six-well plates, to allow the use of a Luer forceps to get smaller pieces, which were transferred to 25 cm^2^ cell culture flasks with Dulbecco’s modified essential medium nutrient mixture F-12 (DMEM/F-12. Sigma Aldrich, St. Louis, MO, USA), with 20% fetal calf serum (FCS, Gibco, Carlsbad, CA, USA) and 1% penicillin/streptomycin (P/S) (Invitrogen, Carlsbad, CA, USA) at 37 °C and 5% CO_2_. The medium was changed every day, and, after a monolayer, confluent cells were checked for phenotype and preserved cryogenically. For the present study, cells from three different donors were used up to passage 3 [18,19].

#### 2.3.2. Cell Culture Experimentation

Human primary osteoblasts from cryogen were first defrosted in 25 cm^2^ cell flasks for 7 days or until confluence was obtained. After reaching confluence, cells were detached with trypsin and suspended in DMEM/F-12 with 10% FCS and 1% P/S, while osteoblasts were counted with a Neubauer counting chamber and set up to a concentration of 1.5 × 10^4^ cells/mL. Bovine bone block (B) samples were standardized into cylinders of 2 mm radius and 2 mm height, and bovine bone granule (G) samples were standardized to a volume of 0.25 cc. Samples were prepared and placed in a 48-well culture plate under sterile conditions. One milliliter of cell suspension (1.5 × 10^4^ cells/mL) was seeded on top of blocks (pOB + B) and on top of granules (pOB + G) in duplicates. In addition, 1 mL of cell suspension without biomaterial (pOB) and biomaterial with 1 mL of culture medium without cells (B and G) were used as control groups. After 24 h of cultivation at 37 °C in a humidified atmosphere, biomaterials were transferred to new wells with fresh culture medium to evaluate only the attached cells. Assay plates were cultured at 37 °C and 5% CO_2_ for 7 days. The culture medium was changed, and the supernatant of culture was collected after 24 h, 72 h, and 7 days of cultivation. A lactate dehydrogenase (LDH) assay and an enzyme-linked immunosorbent assay (ELISA) were performed in duplicate for supernatants from the different experimental groups at three different timepoints (24 h, 72 h, and 7 days). Histology and immunofluorescence staining were performed for samples after 24 h and 7 days.

#### 2.3.3. LDH

LDH assessment was performed in duplicate for the different supernatants gained at three different cultivation timepoints (24 h, 72 h and 7 days) using the Pierce LDH cytotoxicity assay kit (Thermo Fisher Scientific, Rockford, IL, USA); the assay was performed according to manufacturer’s instructions, and absorbance was measured with a microplate reader (Infinite M200, TECAN, Grödig, Austria) set to 490 nm wavelength with a 680 nm correction reference reading.

#### 2.3.4. ELISA

Supernatants from test and control groups were collected and replaced at 24 h, 72 h, and 7 days of cultivation. The concentrations of cytokines and growth factors TGF-β1, TNF-α, IL-1β, IL-6, OPG, and IL-8 were assessed using DuoSet^®^ ELISA Development Systems (R&D Systems, Minneapolis, MN, USA) following the manufacturer’s instructions. All samples were measured in duplicate with a microplate reader (Infinite M200, TECAN, Grödig, Austria) set to 450 nm wavelength with a 570 nm correction reference reading. Outputs were plotted as concentration estimates by interpolating a parameter logistic curve against standard in Graph-Pad Prism version 8.0 (Graph-Pad Software). 

#### 2.3.5. Immunofluorescence

Samples of tests and controls were stained for osteopontin (OPN) to evaluate the adhesion of osteoblasts at the material surface. Rabbit anti-human OPN (1:200 in 1% bovine serum albumin/PBS) was used as the primary antibody. Samples previously fixed in Histofix^®^ (AppliChem, Darmstadt, Germany) were washed three times with PBS before permeabilization with 0.5% Triton-X/PBS. Permeabilized samples were washed again three times in PBS and incubated with the primary antibody for 1 h at room temperature. The washing step was repeated three times after incubation before samples were treated with secondary anti-rabbit Alexa Fluor 546 antibody (goat; Invitrogen, Molecular probes, Waltham, MA USA) diluted 1:1000 in 1% BSA/PBS for 60 min in the dark at room temperature. The cell nuclei were counterstained with DAPI (Invitrogen, Molecular probes, USA), using a 1:400 dilution of 0.5% DAPI stock solution.

### 2.4. Statistical Analysis

All statistical analyses were performed in GraphPad Prism (GraphPad Prism version 8.0.1, GraphPad software Inc., San Diego, CA, USA) considering a 95% confidence level (*p* < 0.05) and a beta error of 20%. The average results at each timepoint were compared by two-way ANOVA with Tukey’s post hoc test for multiple comparisons.

## 3. Results

### 3.1. Biomaterial Characterization Ex Vivo

#### 3.1.1. Histological Evaluation

**Figure 1:**
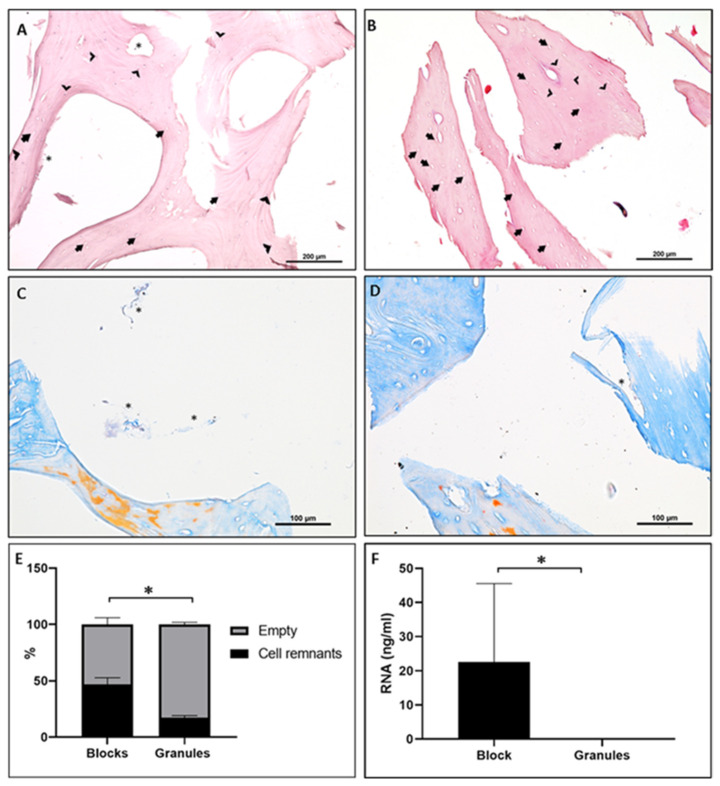
(**A**,**B**) Ex vivo H & E staining and histomorphometry of DBBM block (**A**) and granules (**B**) at 20× magnification. (**C**,**D**) Ex vivo Azan trichrome staining of bovine bone blocks (**C**) and granules (**D**) at 40×. Arrows indicate empty osteocyte lacunae and arrow heads indicate examples of osteocyte lacunae with cell or organic remnants of bovine tissue inside; asterisks mark organic or connective tissue-like remnants outside the lacunae. (**E**) Ratio between empty osteocyte lacunae and lacunae presenting cell remnants. (**F**) Total amount of RNA, assessed with NanoDrop (NanoDrop, Wilmington, NC, USA), for block and granules. * Statistical significant difference (*p* < 0.05).

In the bone block (B) group, a lamellar structure was present in the demineralized sample, with thin trabeculae and interconnected pores. Additionally, the samples exhibited a morphology similar to human bone, but with increased porosity. In H&E staining, organic remnants were present inside the Haversian canals and in the margin of trabecula, indicating possible fragments of periosteum not removed by the chemical baths (Figure 1A,B). Furthermore, regarding all tested lot numbers of bone blocks (B), on average, 45.55% (±7.12) of osteocyte lacunae presented cellular remnants (Figure 1A,C,E). 

For the tested lot numbers of granules (G), a lamellar structure including osteocyte lacunae was observed. Cell-like structures were documented within the osteocyte lacunae. The quantitative analysis of the granules showed, in 17.31% (±1.31) of lacunae, the presence of some traces of organic remnants (Figure 1E).

For both materials, Azan staining evidenced a mature mineralized bone with the presence of possible collagen remnants and connective-like tissue. There were Haversian canals without any organic remnants, although others presented connective-like tissue fragments (Figure 1C,D). No TRAP-positive cells were found in any of the examined batches (data not shown).

#### 3.1.2. Molecular Analysis

In three of five batches of Bonefill^®^ Porous Block, it was possible to find and isolate RNA. RNA amounts ranged from 14.4 to 47.7 ng/µL, with a 260/280 rate of purity of 1.76 to 1.94 (Figure 1F). In the samples of Bonefill^®^ Porous Granules, no trace of RNA was found (Figure 1F).

### 3.2. Human Osteoblast (pOB) Response In Vitro

#### 3.2.1. Growth Factor and Cytokine Release

**Figure 2:**
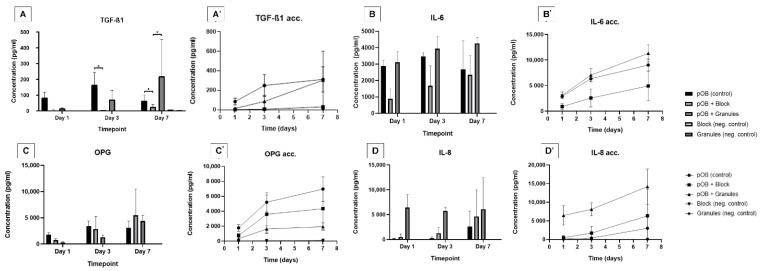
Cytokine concentrations in the supernatant of deproteinized bovine bone matrix (DBBM) in block or granules compared to control layer culture at different cultivation timepoints. (**A**) TGF-β1 concentration in the supernatant. (**A′**) Accumulated values for TGF-β1 concentration kinetics in the supernatant. (**B**) IL-6 concentration in the supernatant. (**B′**) Accumulated values for IL-6 concentration kinetics in the supernatant. (**C**) OPG concentration in the supernatant. (**C′**) Accumulated values for OPG concentration kinetics in the supernatant. (**D**) IL-8 concentration in the supernatant. (**D′**) Accumulated values for IL-8 concentration kinetics in the supernatant. Results for TNF-α, IL-1, and IL-17 were under the range of measurement. * Statistical difference (*p* < 0.05; two-way ANOVA and Tukey’s post hoc test).

The release of TGF-β1 in the group of cells cultured on bone granules (pOB + G) increased at timepoint 2 (day 3) and was higher compared to release of TGF-β1 in the group of cells cultured on the bone block (pOB + B). After 7 days, the TGF-β1 secretion by pOB + G (218.85 ± 234.62 pg/mL) was higher than that in both pOB + B (24.34 ± 15.59 pg/mL) and control (62.6 ± 39.55 pg/mL) (*p* < 0.05) (Figure 2A). For IL-6 and OPG release, there was no statistical difference between experimental groups; for both cytokines, there was a general increase inOPG production from 24 h to 7 days in the test groups pOB + B and pOB + G (Figure 2B,C). The IL-8 secretion was higher in pOB + G (6.49 ± 2.57 ng/mL), in comparison to pOB + B and control, at 24 h. IL-8 release increased in all groups over the timepoints, but the control group presented lower IL-8 expression at 7 days (2.64 ± 3.04 ng/mL) compared to both pOB + g and pOB + B.

Considering the kinetics of cytokine release during the study period, all groups presented a similar increasing pattern of IL-6, IL-8, and OPG concentrations (Figure 2). Regarding TGF-β1 release the pOB + B presented the lowest amount of TGF-β1 secretion at the end of the experiment on day 7 (30.22 ± 14.94 pg/mL, *p* < 0.05). pOB + G presented the highest increment of TGF-β1 from 72 h to 7 days (85.68 ± 62.89 pg/mL to 304.53 ± 295.63 pg/mL, *p* < 0.05) (Figure 2A′). IL-6 release in pOB + G supernatants was higher than in pOB + B supernatants after 72 h of cultivation (7.5 ± 1.32 ng/mL vs. 2.6 ± 1.78 ng/mL, *p* < 0.05) and 7 (11.31 ± 1.67 vs. 4.91 ± 2.91 ng/mL, *p* < 0.05), but similar to control (9.01 ± 1.18 ng/mL, *p* > 0.05) (Figure 2B′).

The pOB + G IL-8 cumulative secretion (8.12 ± 1.74 ng/mL) was higher than both control (0.39 ± 0.34 ng/mL) and pOB + B (1.72 ± 1.80 ng/mL), for the entire evaluated cultivation period. In the pOB + G group (14.21 ± 4.74 ng/mL), higher IL-8 secretion can be observed compared to the control (3.03 ± 3.38 ng/mL, *p* < 0.05), but this was similar to IL-8 concentration in the pOB + B supernatants (6.38 ± 7.13 ng/mL, *p* > 0.05) (Figure 2D′).

After 24 h, OPG in supernatants of the control group (1.78 ± 0.45 ng/mL) was higher than in both pOB + B (0.75 ± 0.26 ng/mL, *p* < 0.05) and pOB + G (0.31 ± 0.14 ng/mL, *p* < 0.05) supernatants. After 7 days, OPG secretion in pOB + G (1.35 ± 1.24 ng/mL) remained lower than in the control (5.87 ± 3.46 ng/mL, *p* < 0.05), but OPG in pOB + B (4.29 ± 2.85 ng/mL) did not differ from the control (*p* > 0.05).

#### 3.2.2. Histological and Immunofluorescence Evaluation

**Figure 3:**
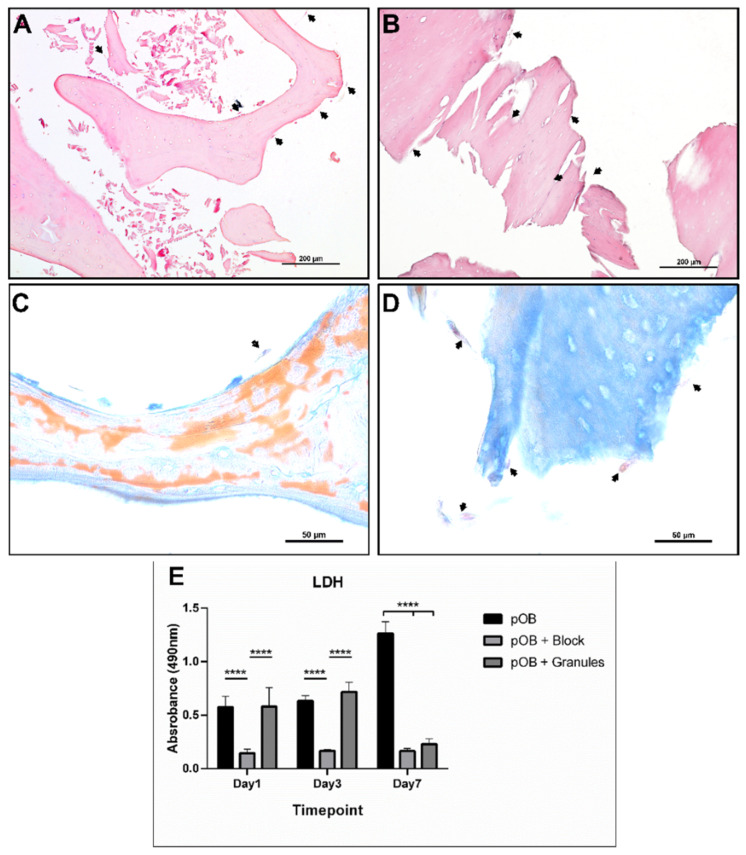
Histologic samples of DBMM in block and granules after 7 days with cultivated primary human osteoblast (pOB) cell culture. (**A**) pOB + block after 7 days, H&E 20×. (**B**) pOB + granules after 7 days, H&E 20×. (**C**,**D**) Azan trichrome staining of bovine bone blocks (**C**) and granules (**D**) 100×; arrows indicate examples of pOB on material surface. (**E**) LDH determination of different experimental groups at the three different timepoints. **** Statistical difference (*p* < 0.01).

**Figure 4:**
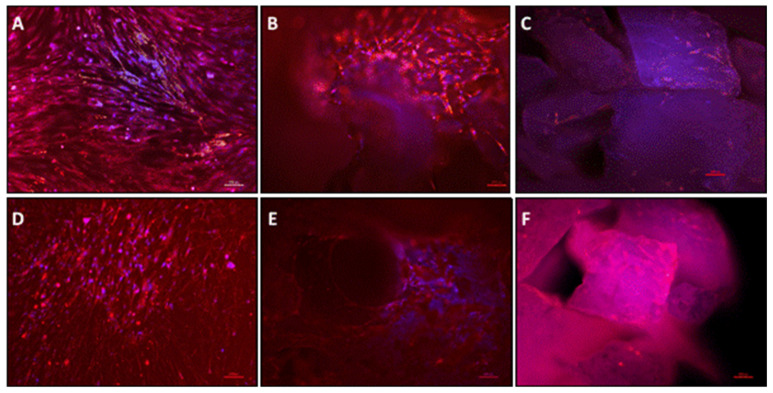
Osteopontin immunofluorescence staining and DAPI nuclear staining for osteoblasts at 200× magnification. (**A**) Primary human osteoblasts (control) at day 1 cultivated on culture plastic. (**B**) Surface of bovine bone block cultured with pOB on day 1. (**C**) Bovine bone granules cultured with pOB on day 1. (**D**) pOB on day 7 (control). (**E**) Surface of Bovine bone block cultured with pOB on day 7. (**F**) Bovine bone granules cultured with pOB on day 7. Scale bars = 100 µm.

Samples were fixed and prepared for histological analysis after 24 h and 7 days of in vitro cultivation. An increase in the number of cells adhered to the material surface was observed in both materials on day 7 (Figure 3A,B), with cells penetrating the material lacunae. Azan-stained cells after 24 h of cultivation were well distributed on the material surface of granules, but fewer cells attached to the block’s surface (Figure 3C,D). However, after 7 days of cultivation, it was possible to observe cells filling the lacunae of both tested materials. The cell viability assessed by LDH showed no difference between the pOB control group and pOB + G. However, both the pOB control group and pOB + G showed significantly higher LDH release compared to pOB + B. This pattern was observed on days 1 and 3 of cultivation. Interestingly, after 7 days, the LDH release was the highest in the pOB control group, which was statistically significant when compared to the other two groups. Samples were also stained for osteoblastic marker osteopontin (OPN) via immunofluorescence, and osteoblasts were positively marked on both material surfaces on days 1 and 7 (Figure 4). In bone blocks, osteoblasts were mostly located in the lamellar structure (Figure 4E).

## 4. Discussion

Biomaterial purification has been an issue in the field of bone substitute materials since its conception [20]. Non-autologous bone substitutes from different origins, either from same species donors (allografts) or different species (xenografts), carry the potential of disease transmission or even the induction of an adverse immunological reaction [9,16,21]. These materials can contain bacteria, viruses, antigens, immunological molecules, and proteins that ideally should be eliminated or inactivated by standardized procedures before clinical application [8,16,22,23].

Several techniques have been purposed to eliminate organic remnants from non-autologous grafts, and standardized methods have been published and validated in the past 20 years [16,21]. In particular, for bovine-derived materials, there is a strong concern about Creutzfeldt–Jakob disease (CJD) in humans, as well as bovine spongiform encephalitis (BSE). In Europe, a publication by the German Ministry of Health dated 1994 and reviewed in 1996 (Bundesgesundheitsamt, 1996) [24] stated that bone substitute materials regained from sources such as bovine, caprine, or sheep must aim to minimize the risk of BSE transmission to humans. According to this, the material should be evaluated by six parameters: (1) origin and feeding of the animals; (2) type of tissue used for production; (3) processing steps for inactivation of prions; (4) amount of raw material needed to produce one daily dose; (5) number of daily doses; (6) method of application. Each parameter is classified according to a logarithmic scale. Higher numbers indicate a lower risk of infection. The origin and feeding of the animals is a strong parameter. In this study, analyzed materials were gained from tracked Brazilian cattle, free of BSE (according to the manufacturer), which minimizes the risk of prion transmission, while bone tissue is classified, as suggested above, as the lowest risk tissue for prion contamination. According to these parameters, the tested biomaterial achieves a score of 22 points, over the 20 points necessary to be considered safe for CJD transmission.

For parameter 3 (processing steps for inactivation), several methods of purification have been described in the literature, but it is known that heating above 1000 °C is the most effective way of protein denaturation [17,21]. However, special attention has been given to purification procedures that do not affect the ultrastructure, as well as the mechanical and osteoconductive properties, of materials [16]. High-temperature heating reduces the material porosity and melts the lamellar structure, impairing material wettability and osteogenic cell attachment. On the other hand, the manufacturer of the tested materials states that Bonefill^®^ is submitted to a “sequence of baths that solubilize the organic structures such as cells remaining from the organic matrix, fibers, and proteins”, and it is processed at low temperature [25,26]. 

Some previously evaluated bovine-derived bone materials, such as Bio-OSS^®^, are considered to be free of organic residues [8], and their purification procedure consists of an initial bath for fat and gross organic residue removal, followed by heating up to 300 °C before a highly alkaline solution bath (pH > 13). Previous studies proved that Bio-OSS^®^ is free of organic remnants [8,21,27]. However, this process results in a loss of resistance and mechanical properties; furthermore, this material when presented in blocks usually cannot be fixed to bone with screws.

The bone block evaluated in this study preserves the macro lamellar structure similar to human bone, with mature mineralized interconnected trabecula and high porosity, which has been reported to promote good mechanical resistance. Clinical studies showed that the herein used bone block preserves good mechanical stability, which allows the successful use of screws to fix the Bonefill^®^ blocks to the jawbone of patients [26,28]. However, the exclusive chemical processing is not efficient in removing all organic material from bovine blocks, whereas it appears to be more effective in granules. These differences between granules and blocks can be related to the three-dimensional structure of the blocks which could prevent a total penetration of the chemicals within the deeper parts of the bone block.

In a previous ex vivo study, Orlowska et al. [7] identified the presence of a lamellar structure and cellular remnants in a xeno-synthetic DBBM, with intentional collagen addition. According to the authors, collagen-containing bone substitutes could provide stability to bone formation and improve the biomaterial results. However, more studies are necessary to evaluate the biocompatibility of bone substitute materials containing organic remnants [7]. 

Most available bone substitute materials contain some degree of impurity, and it is not clear how these features interfere with the biocompatibility and bone formation. Inflammatory foreign body reaction is a current concern regarding the use of biomaterials for bone augmentation, and the pattern of macrophage reaction seems to be an important marker to predict if a biomaterial becomes successfully incorporated into host bone and allows new bone formation, or if it may undergo complete encapsulation [9,13,29,30].

Previously to in vitro evaluation, the tested batches of bovine bone block presented a mean amount of detectable RNA of 22.6 ng/µL, ranging from 14.4 to 47.7 ng/µL, compared to no RNA detected in granules. The cell culture of primary human osteoblasts on the surface of both materials aimed to identify whether the difference in xenogeneic genetic material content could interfere with the immune response of human osteoblasts in vitro; thus, the cell culture supernatants were screened for proinflammatory cytokines such as TGF-β1, IL-1, and IL-6 and osteoprotective molecules such as OPG using an enzyme-linked assay. 

Moreover, the adherent cells and the cell viability were assessed by LDH expression. These results need careful interpretation. In our study, the pOB-seeded granules and blocks were moved to a new tissue culture plate after 24 h to ensure that the performed tests were only related to cells adherent on the biomaterial surface and not those attached to the plate. Therefore, we assumed that the number of cells adherent to the granules and blocks after sample transfer was lower than in the pOB control group. This assumption was supported by the observation of the low number of adherent cells as shown by histology and immunofluorescence (Figure 3 and Figure 4). This observation may, thus, explain the observed LDH expression in DBBM block and granules groups after 7 days. This lower quantity of adherent cells in the tested material could also be related to lower cytokine expression. However, one limitation of the present study is the lack of quantification of adherent cells, which was technically very challenging. Therefore, further studies are needed to provide more precise data on this point.

In our study, the used bone block, containing organic remnants, did not induce a pronounced inflammatory reaction in human osteoblasts, compared to the DBMM granules without traces of RNA or cellular residuals. However, the secretion of TGF-β1 in the group pOB + B was significantly lower than in the group pOB + G at all timepoints, which could be explained by the lower number of cells attached to the material surface in the pOB + B group. TGF-β1 is commonly secreted by osteogenic cells, and it is considered a pleiotropic interleukin in bone, which usually induces bone formation by stimulating OPG and inhibiting the RANK pathway, leading to osteoid formation [31,32]. Bellone et al. [33] performed an in vitro study with human bone marrow cells seeded on implants coated with equine-derived bone granules and identified that the release of cytokines from the TGF-β1 family increased 1.5-fold compared to noncoated implants. In our study, the bovine derived bone in granules also promoted a slight increase in TGF-β1 release, which can be related to the hypothesis that the particle size and the shape of biomaterials are determinant for cell adherence and cytokine release [34,35,36,37]. In terms of the results obtained, an important limitation of our study is the use of primary human osteoblasts to evaluate immunologic response; hence, further studies must be conducted using monocyte or macrophage cell cultures to better compare the antigenic properties of different bone substitute presentations. Furthermore, a comparison between study methods should be performed, to evaluate the need of changing the culture material after the first 24 h.

The change of culture medium could result in a reduced cell count at the material surface. In our study, we attempted to perform DNA quantification to estimate the difference in cell count between control and test groups (see Supplementary Materials); however, due to the small sample size, we could not identify a statistical difference in the DNA amount between groups. Therefore, it was not possible to adjust the cytokine secretion to the cell count.

IL-6 is a proinflammatory cytokine naturally released by osteoblasts; its functions in bone homeostasis are related to osteoclast activation and osteoblast maturation. Moreover, it was shown to be more highly released by immature than mature osteoblasts. Amerio et al. [37] evaluated the secretion of IL-6 by osteoblasts exposed to DBBM, presenting a 3–5-fold lower release in the group exposed to DBBM compared to control. In our study, we did not observe a significant difference in the IL-6 expression between groups; furthermore, none of the tested materials induced a significant increase in this cytokine. Bellone et al. [33] also investigated the effect of coating titanium implants with equine-derived bone and evaluated the cytokine release of human bone cells, but they did not find any significant increase in IL-6 expression.

The OPG expression did not differ between the evaluated groups. However, an increase in OPG release over time was observed in all analyzed groups. Similarly, Kubosch et al. [10] observed a twofold increase in OPG in vitro expression of human osteoblasts seeded on DBBM granules in comparison to cells seeded on human cancellous bone. OPG, together with RANK/RANKL, represents the main control mechanism for osteoclastogenesis. OPG is a cytokine receptor expressed by osteoprogenitor cells and osteoblasts to inhibit the differentiation of osteoclasts and to control the bone resorption; thus, the rise in OPG concentration could be related to bone anabolic metabolism [10,38,39].

IL-8 was also higher for pOB + G compared to the control and pOB + B; this cytokine is particularly related to particle-induced chemotaxis and the recruitment of neutrophils at the early stage of the inflammatory response [34]. It was previously demonstrated that high-temperature sintered biomaterials induce more IL-8 production and a greater reaction of polymorphonucleated cell (PMG) [34,35,40]. Osteoblasts are potent inflammatory mesenchymal cells. Beyond their native osteogenic function, they also have a cytokine-releasing function when stressed, acting as a modulator of the bone immunologic response [14,32]. Velard et al. [36] demonstrated that HA crystals induce a higher release of IL-8 and IL-1 when cultured with polymorphonucleated cells. Their release was increased 3.75–6-fold in comparison to the control culture. These results are similar to the findings of the present study demonstrated by DBBM granules, especially in the first 24 h.

The applied methods in this study were unable to document an adverse immune response; therefore, within the limitations of the present study, it is not possible to validate the use of impure materials including organic residues. the possible long-term complications of this impurity are also unclear. According to Fretwurst et al. [41], the presence of organic remnants is related to the expression of class I and II major histocompatibility complex (MHC) molecules, which are responsible for antigen-antibody interaction; the MHC incompatibility of donor and host can activate the lymphocyte response, leading to an antibody reaction. It is also important to emphasize that, in the same study, Fretwurst et al. [41] did not find MHC traces in the gold standard xenografts, free of organic remnants.

For both types of DBBM tested, it was possible to identify the accumulation of osteoblasts at the material surface. In the immunofluorescence images in day 1, the cells appeared to be at the top of material, whereas, on day 7, the accumulation could be observed toward the porous structure, which was also documented by at histological staining.

Our results suggest that both tested materials induced a similar inflammatory response by osteoblasts, despite the differences in the presentation (block or granules) and purity degree. The results of monoculture cells limit the extrapolation of results, and in vivo inflammatory reaction studies should be performed to evaluate biomaterials with different purification degree, while also comparing different commercial brands.

## 5. Conclusions

The presence of organic remnants in the tested materials did not induce an adverse inflammatory response in human primary osteoblasts. However, within the limitations of the presented study, it is not possible to state whether the use of xenogeneic biomaterials containing organic remnants is safe. Thus, the implications and adverse effects of their implantation are still not understood. Furthermore, the presentation form of DBBM seems to be more important to human primary osteoblasts.

## Data Availability

The data presented in this study are available on request from the corresponding author.

## Supplementary Materials

The supporting information can be downloaded at: https://www.mdpi.com/article/10.3390/ma15030999/s1.
