# Supplementary figures and images for: Cellular Response of Human Osteoblasts to Different Presentations of Deproteinized Bovine Bone
Source: Materials (Basel). 2022 Jan 27;15(3):999. doi: 10.3390/ma15030999 (PMC8839050; doi:10.3390/ma15030999)

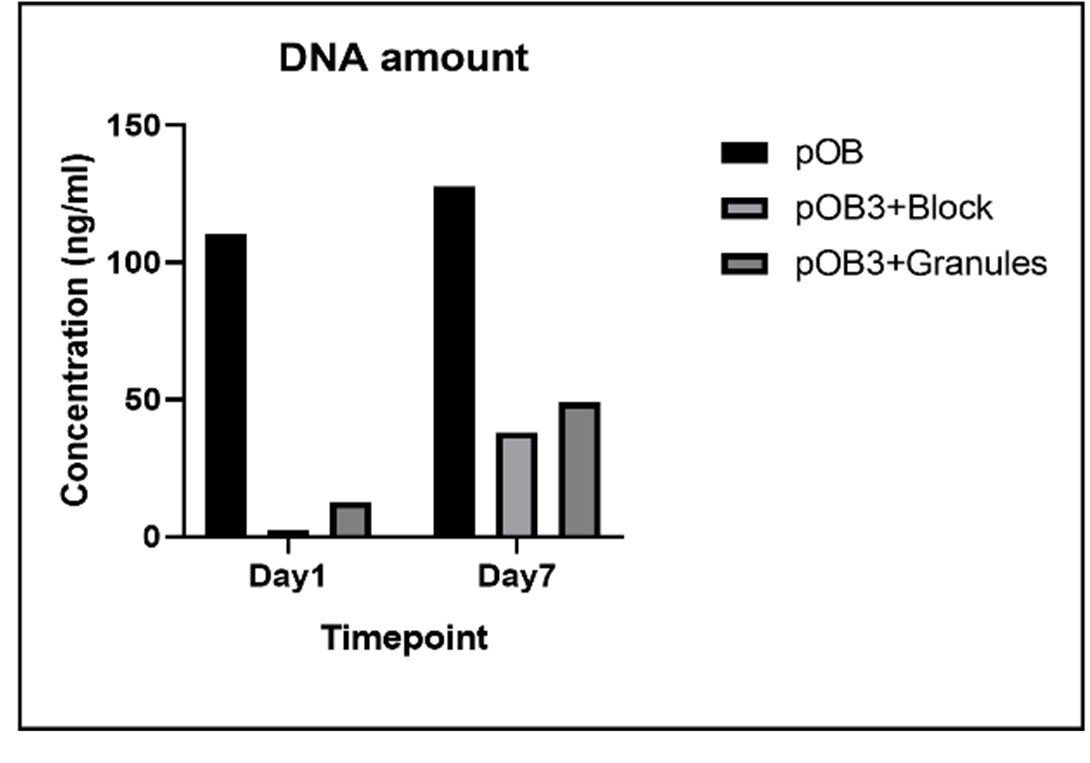

Supplement: Supplementary file 1 [file materials-15-00999-s001.zip › materials-1483070-supplementary.png]
